# Applications of cell- and tissue-specific ‘omics to improve plant productivity

**DOI:** 10.1042/ETLS20210286

**Published:** 2022-03-16

**Authors:** Bhavna Hurgobin, Mathew G. Lewsey

**Affiliations:** 1 La Trobe Institute for Agriculture and Food, Department of Animal, Plant and Soil Sciences, School of Life Sciences, La Trobe University, AgriBio Building, Bundoora, VIC 3086, Australia; 2 Australian Research Council Research Hub for Medicinal Agriculture, La Trobe University, AgriBio Building, Bundoora, VIC 3086, Australia

**Keywords:** cannabis, germination, network, seed, transcriptomics, trichome

## Abstract

The individual tissues and cell types of plants each have characteristic properties that contribute to the function of the plant as a whole. These are reflected by unique patterns of gene expression, protein and metabolite content, which enable cell-type-specific patterns of growth, development and physiology. Gene regulatory networks act within the cell types to govern the production and activity of these components. For the broader organism to grow and reproduce successfully, cell-type-specific activity must also function within the context of surrounding cell types, which is achieved by coordination of signalling pathways. We can investigate how gene regulatory networks are constructed and function using integrative ‘omics technologies. Historically such experiments in plant biological research have been performed at the bulk tissue level, to organ resolution at best. In this review, we describe recent advances in cell- and tissue-specific ‘omics technologies that allow investigation at much improved resolution. We discuss the advantages of these approaches for fundamental and translational plant biology, illustrated through the examples of specialised metabolism in medicinal plants and seed germination. We also discuss the challenges that must be overcome for such approaches to be adopted widely by the community.

## Introduction

Plants have been used continuously in a wide array of foods, medicines, textiles and construction materials since their domestication ∼10 000 B.C. [1,2]. However, it was only after the nineteenth century that major scientific breakthroughs were made in plant breeding, driving significant advances in productivity. The development and application of ‘omics technologies in recent years, such as high-throughput sequencing, proteomics and metabolomics, have enhanced research into plant biology tremendously [3–7]. As a result, we have gained a better understanding of the genetics and complex biological processes that underpin plant productivity. These discoveries have been translated to real-world agriculture through improved breeding practices and biotechnology solutions that have increased yield [8–10].

Plant tissues consist of many different cell types that each have specific functions. These functions are driven by distinct biochemistry, physiology and gene expression programmes. The concerted activity of cell types determines the properties of plant tissues and organs but their correct function requires careful coordination and integration of signalling networks [3,5,11–15]. ‘Omics technologies have been applied extensively to determine the mechanisms controlling the accumulation of proteins and metabolites within organ and tissue types [4,6,16,17]. Recent advances in single-cell resolution ‘omics methods, such as single-cell RNA-seq (scRNA-seq), have further advanced our ability to understand spatiotemporal responses to biological stimuli and compartmentalisation of functions [11–15]. These methods are not yet mature or widely adopted but widespread enthusiasm exists amongst the plant science community. In this respect, the Plant Cell Atlas (PCA) framework has been initiated to overcome the knowledge gaps and technical challenges that stand in the way [18,19].

Cellular function is closely linked with spatial location [20]. Consequently, we need to understand both expression of gene products at the single-cell level and the spatial context of cells. In other words, the knowledge of where and how closely individual cells are located relative to one another in native tissues is required to understand the intercellular communication that occurs upon biological stimulation. However, the isolation of single cells for single-cell analysis methods, such scRNA-seq, typically requires tissue dissociation and so loses this spatial information. Spatial transcriptomics overcomes this issue by physically localising gene expression in distinct areas of tissues [21]. The method can be considered analogous to classic RNA *in situ* methods but assays complete transcriptomes rather than individual transcripts. This approach can be combined with scRNA-seq to survey the expression of gene products of distinct cellular subpopulations within intact plant tissues [20].

**Figure 1.**
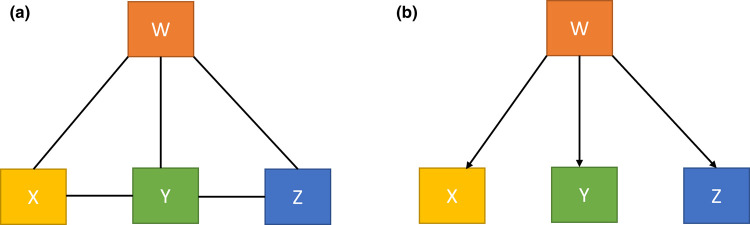
Simplified models depicting gene networks used in biological research. Coloured boxes represent genes or transcription factors (nodes) and black lines/arrows represent the presence of a connection between nodes (edges). (**a**) In a gene co-expression network, genes W, X, Y and Z have similar patterns of expression, and are therefore connected. However, this type of network does not meaningfully reflect how these genes interact. (**b**) In a gene regulatory network, edges have directionality and depict causal relationships between nodes. For example, W, X, Y and Z are co-expressed and correlated. However, X, Y and Z do not interact with each other even though they are regulated by W.

Individual, gene-by-gene analysis is common in plant science research, but does not provide the holistic, systems level view of biological processes needed to understand the organism as a whole [22,23]. This realisation has given rise to the concept of gene networks as shown in Figure 1 [22,23]. The underlying principle of this powerful approach is that a biological system can be represented as a network graph of interacting genes or gene products [24]. Gene co-expression network analysis has been applied extensively to plant tissues to identify genes with co-ordinated expression patterns during development or exposure to environmental cues (Figure 1a) [25–27]. Similarly, gene regulatory network analysis has been employed to decipher the hierarchical relationship between transcription factors, their target genes and associated signalling pathways (Figure 1b) [23,28]. Extension of these approaches to the analysis of cell types at single-cell resolution has begun and will provide a clearer, deeper understanding of the regulatory programmes that underpin cellular processes [29–31].

In this review, we provide an overview of how ‘omics technologies have been applied to study plant tissues, illustrating this through two major examples; specialised metabolism in medicinal plants, and seed germination in cereal crops. We highlight knowledge gaps, provide insight into the types of analyses that could be performed at the single-cell level and the biological insight this would enable, and discuss how network theory and spatial transcriptomics could be leveraged. We also discuss the challenges that must be overcome along the way.

## Glandular trichomes in cannabis and the production of cannabinoids

**Figure 2.**
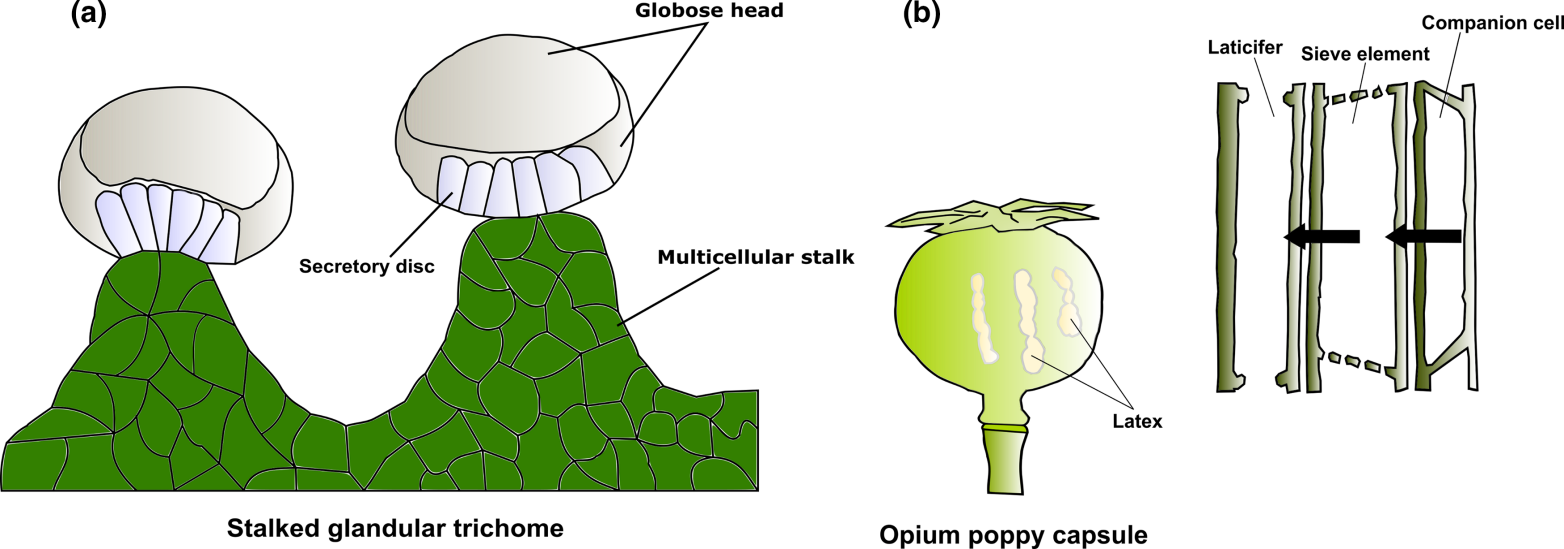
Site of specialised metabolites synthesis, accumulation and storage in *Cannabis sativa* and *Papaver somniferum* (opium poppy). (**a**) The stalked glandular trichome of cannabis consists of a globose head which protrudes from the epidermal surface via a multicellular stalk. The head region contains several secretory disc cells that produce and store specialised metabolites such as cannabinoids and terpenes. (**b**) The opium poppy capsule wall contains an extensively branched network of companion cells, sieve elements and laticifers where latex exclusively occurs. Latex represents the site of alkaloid accumulation in poppies.

Phenotypic plasticity is a unique and defining property of plants. Plants are immobile and therefore must adapt to the range of dynamic local environmental conditions, rather than move to escape them. Challenges they face include wide daily temperature variation, water and nutrient availability, pathogen and animal attack [32–36]. Alterations of metabolism are an important component of adaptation mechanisms [37]. Accordingly, plants produce a plethora of specialised (also known as secondary) metabolites via complex metabolic pathways. These pathways are dynamic and can quickly alter the profile of specialised metabolites thereby enabling plants to adapt and mediate environmental interactions [37,38]. Many specialised metabolites are produced and accumulate to high levels in particular cells or organs that have unique properties (Figure 2) [39–41].

*Cannabis sativa* exhibits remarkable specialised metabolite diversity and is currently the focus of much attention from the pharmaceutical industry, breeders and researchers. It is a prolific producer of cannabinoids and terpenes in specialised structures called capitate stalked glandular trichomes (Figure 2a) [25,42]. Over 120 cannabinoids have been identified, amongst which Δ^9^-tetrahydrocannabinol and cannabidiol are the most abundant and of primary interest to the community [43,44]. More than 100 terpenes have been reported, but are largely understudied compared with the cannabinoids [42,45]. Proteomics has been successfully used to identify several hundreds of proteins in individual cannabis tissues, including the glandular trichomes, some of which are involved in the biosynthesis of secondary metabolites [46,47]. However, there is remarkable variation in secondary metabolite profiles between cannabis cultivars/strains [48,49]. The mechanisms underlying this might be determined by comparing the glandular trichome-specific proteomes of cannabis cultivars that have different metabolite profiles (termed chemotypes). The integration of such studies with tissue-specific transcriptomics and metabolomics analyses, as well as cultivar-specific therapeutic or recreational user experiences, could reveal the broader regulatory mechanisms of secondary metabolism and identify uncharacterised minor compounds that have unexplored therapeutic or industrial benefits.

The glandular trichome is an excellent model to study cell-specific gene regulation. It is composed of a multicellular stalk and a globose head of secretory cells where metabolites are synthesised and accumulate [25]. Transcriptomics, gene co-expression network analysis and metabolomics have been applied to the cells of glandular trichomes, identifying genes associated with the production of major compounds that are primarily or solely active in these cell types [25,26,45,50]. Glandular trichomes were enriched by mechanical means then comparisons made to different cell types, which allowed trichome-specific processes to be identified [25,26,47]. Gene regulatory network analysis is an ideal way to extend these studies. It would enable the identification of transcription factors and their target genes that could be used in breeding programmes or in biotechnology applications. The function of gene regulatory network components can be examined and manipulated using techniques such as gene editing [51,52]). Functional characterisation through *in vivo* mutational analysis would help validate the relationships between network components and the phenotypes that the networks influence. The effect of transcriptional regulators within networks might also be investigated at single-cell resolution using high-throughput *in vitro* approaches, for example, targeted Perturb-seq (TAP-seq) [53,54]. It is also possible to reconstruct parts of gene regulatory networks in heterologous systems, such as yeast, to identify the essential constituents and core parameters of these networks [55]. This synthetic biology approach has successfully been applied in yeast to study plant hormone signalling pathways and the same principle could be used for cannabis to recapitulate specialised metabolism pathways in heterologous systems [56,57].

Transcription factors and genes that drive cannabis glandular trichome initiation and development, and consequently the production of specialised metabolites, are attractive targets for industrial producers who wish to increase or manipulate yield. Transcription factors that regulate specialised metabolism have been identified in the glandular trichomes of *Artemisia annua* and *Solanum lycopersicum* [58–60]. A recent study by Qin et al. demonstrated that overexpression of transcription factor *AaMYB17* in *A. annua* increased glandular trichome density by 1.3–1.6 fold. Production of the specialised metabolite artemisinin, which is an anti-malarial compound, also increased by 50% [58]. Defining the gene regulatory network surrounding *AaMYB17* would allow us to better understand how it regulates these biological processes, providing both increased mechanistic understanding and additional candidate genes for crop improvement. Methods that identify transcription factor target genes genome-wide, such as DNA affinity purification sequencing (DAP-Seq) and chromatin immunoprecipitation sequencing (ChIP-seq), could be applied to reveal the suite of regulated genes [61–64]. This information could then be related to the genetic and biochemical regulation of specialised metabolism. Transcription factors associated specifically with glandular trichomes in *C. sativa* have also been identified and similarly promising avenues could be followed for the study of cannabinoid and terpenoid biosynthesis pathways [65].

## Tissue-specific localisation of alkaloid biosynthesis and storage in opium poppies

*Papaver somniferum* (opium poppy) is another important medicinal plant. The various products of its benzylisoquinoline alkaloid (BIA) pathway have therapeutic uses including for pain relief, in cancer treatment and as cough suppressants [66,67]. Synthesis and accumulation of BIAs occurs in three distinct cells types of poppy capsules; the companion cells, sieve elements and laticifers (Figure 2b) [68,69]. Summarised, transcription and translation of the majority of BIA pathway genes take place in the companion cells, then the corresponding functional enzymes (NCS, 6OMT, NMCH, 4′OMT, SalSyn, SalR and SalAT) are exported to sieve elements where alkaloids are synthesised. Finally, the alkaloids are transported to the laticifers for storage [68,69]. This has been determined by localisation of expression of individual enzymes in the pathway, using biochemical methods. This individual gene/enzyme approach does not inform us about the surrounding regulatory context, which could be defined using cell and tissue-specific ‘omics studies. Thus far, however, global BIA gene expression has only been investigated at the resolution of organs (leaves, stems, roots, etc.) [70,71]. In future, the isolation of single sieve elements, laticifer cell types and companion cells from a range of poppy cultivars with differing alkaloid contents, and the comparison of cellular gene expression profiles and underlying gene networks among cultivars, would shed more light on the transcriptional regulation differences among opium poppy lineages.

One striking property of opium poppy cultivars is their remarkable variation in alkaloid content and composition. For instance, some plants produce large amounts of morphine and codeine while others synthesise high levels of thebaine or oripavine [72]. This is due to the extensive artificial selection the species has been subjected to which has resulted in genetic variation between cultivars, landraces and lines. Whilst the biochemical properties of many enzymes in the BIA biosynthetic pathway have been defined, comparatively little analysis has been conducted of the effects of natural variation in these enzymes between poppy cultivars nor of the underlying gene regulatory mechanisms and the roles they have in generating this variation [66–71,73]. A recent study by Li et al. [71] determined that the BIA genes were predominantly co-expressed, irrespective of whether they were physically clustered within the genome or not. This finding led to the discovery of uncharacterised genes that may be involved in various stages of the pathway. However, their conclusions were based upon analyses of a single poppy cultivar. The same study demonstrated a link between copy number variation (CNV) of BIA genes and alkaloid production in 10 opium poppy cultivars. This raises the question of how gene CNV can affect the topology of gene networks across genotypes of the same species. Therefore, it would be beneficial to compare cultivar-specific gene networks to have a better understanding of how networks interplay with natural variation underpinning alkaloid production. Comparison of cell-type-specific gene expression profiles and the underlying gene networks among cultivars across developmental stages would allow us to define the regulatory mechanisms controlling BIA variation between different opium poppy lineages.

## Events within individual cells and cell types of germinating cereal seeds

Seed germination is a key step in the life cycle of plants. It is an intricate process that involves a series of morphological, physiological, and biochemical changes that transform the metabolically inactive seed into a highly active seedling [74–76]. Proper timing of germination has a direct influence on crop yield as it ensures that the seedlings have the opportunity to thrive under appropriate environmental conditions [77]. The seed itself is the most valuable product of plants, especially cereal crops, and accounts for more than 60% of food sources around the world [76].

**Figure 3.**
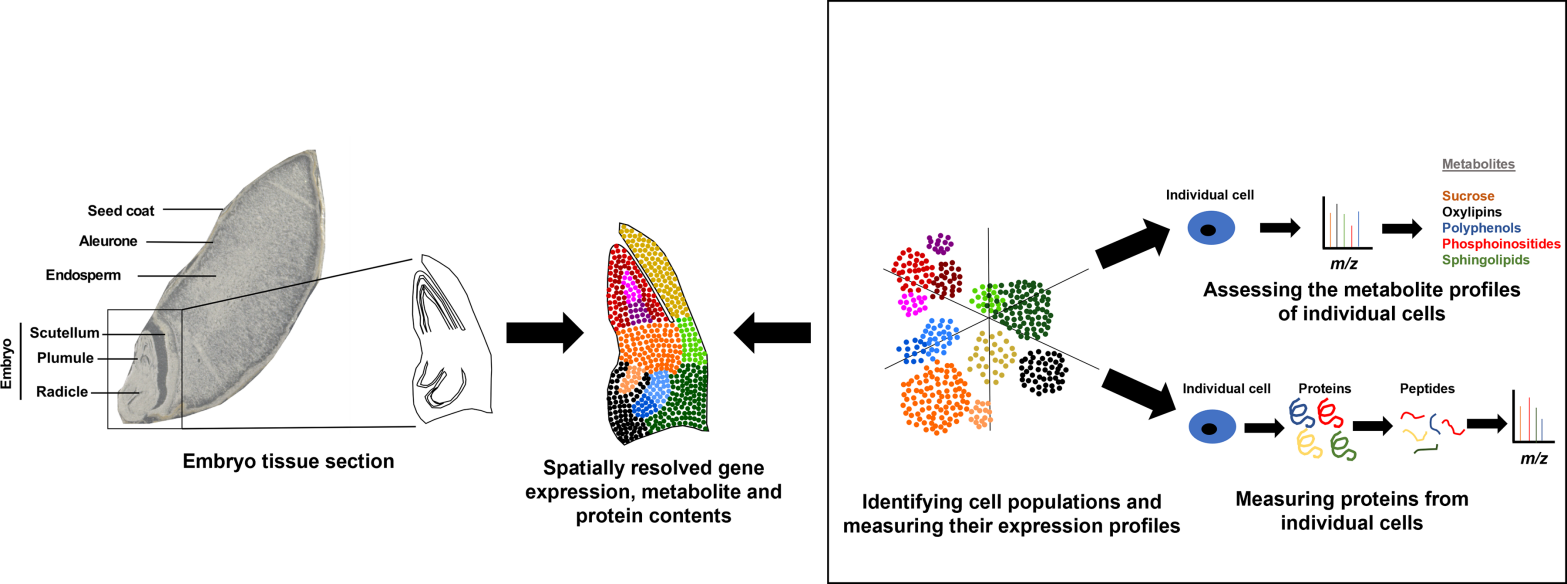
Spatial resolution of single-cell transcriptomics, metabolomics and proteomics in germinating barley seeds. A spatial barcoded map of the barley embryo is obtained using spatial transcriptomics to capture the mRNA transcripts across the embryo tissue section. In parallel, the embryo tissue undergoes tissue dissociation for single-cell RNA sequencing (scRNA-seq), followed by gene expression measurements, dimensionality reduction and clustering of various cell types that constitute the scutellum, plumule and radicle. The single cells are also surveyed for their metabolite and protein contents. Finally, the various single-cell ‘omics measurements are integrated with spatial information to annotate the cell subpopulations [20]. Image adapted from [76] with permission from John Wiley and Sons.

Seed germination has been studied extensively in *Arabidopsis thaliana*, with strong spatial and temporal organisation of growth, development and physiological processes found to exist [78–81]. For example, the decision to break dormancy and proceed with germination is made by small numbers of cells in discreet regions of the radicle, by control of the biosynthesis and perception of the hormones, abscisic acid and gibberellic acid [78]. Specific genes must be activated and repressed at the right times in the various cell types of the seed for this to occur successfully [81]. Similarly, tight spatiotemporal regulation must occur in germinating cereal seeds, but our knowledge of this is extremely limited. A recent study by our laboratory identified co-expressed tissue-specific genes and key TFs at different stages of germination in seeds of barley, an important cereal crop and model for genomics studies of monocot species [76]. We did so by applying laser-capture microdissection (LCM) coupled with RNA-seq. Although LCM can spatially resolve and compartmentalise gene expression, it does so at the bulk tissue level with a minimum resolution of tens of cells [82]. However, cell function and gene expression vary over smaller resolutions than this within the complex structures of the seed [78,80,81]. Resolving patterns of gene expression in single cells of germinating barley seeds and mapping this information onto the spatial location of the underlying cells would, therefore, provide a more specific and comprehensive view of transcriptional activity over time. This would allow us to better understand the functions of known cell types and to identify cell types previously undiscovered. The integration of metabolomics and proteomics within this single-cell spatial transcriptomics framework would enable us to improve structural and functional models of cereal seeds (Figure 3).

Seed germination is strongly influenced by environmental conditions, with abiotic stresses able to have negative impacts [83–85]. One such abiotic stress is soil salinity, which prevents adequate water uptake and drives excessive ion uptake by the seed, leading to toxic effects. This can in turn inhibit or delay seed germination and cause massive yield losses [85]. Unfortunately, the transcriptional regulation of cereal seed germination under salt stress remains poorly understood. Barley is exceptionally salt tolerant relative to other cereal crops and an excellent model for the study of this process [83]. Yousefirad et al. [84] recently investigated the effect of salt stress on seedlings of salt tolerant barley mutants using RNA-seq of bulk tissues. This provided insight into the mechanisms by which established seedlings cope with salt stress, but did not consider the effects of such an exposure during the very early stages of germination nor the cell-type specificity of tolerance mechanisms. In roots, different cell types are essential for tolerance of different abiotic stresses, employing individual strategies to sense and respond [86]. Termed the ‘Gatekeeper Concept’, it is conceivable that similar specialisations exist in seeds of individual cell types to particular stress responses, but this has not been investigated. By identifying these specialisations, defining their roles and characterising the underlying molecular mechanisms, we would be able to better engineer cereal crops with improved performance in challenging environments.

## Challenges of multi-omics and cell-specific strategies for translational plant research

The application of individual omics’ technologies in plant research has undoubtedly been very successful, especially in the last decade [3–7,17,38]. However, each technology comes with its own technical and data analysis challenges and these are further exacerbated at the single-cell level. For instance, each plant cell consists of a polysaccharide cell wall that must be removed to release the cell for scRNA-seq. This occurs via a process called protoplast isolation [12,87,88]. However, plant cell walls differ in thickness and composition across species, tissues, developmental stages and abiotic stresses, making protoplast isolation challenging whenever a new experimental system is examined [12,88]. Furthermore, protoplast isolation can induce unwanted transcriptional or metabolic changes to the cells [12]. This, coupled with the generally larger size of plant cells (100 µm) relative to mammalian cells (10–30 µm) and the variations in cell sizes among plant species, organs and tissues, may prevent some cell types from being captured and profiled by the current, most commonly used single-cell platforms and thereby introduce biases to experiments [12,13,87–90]. Optimised protocols for tissue and cellular dissociation that are applicable to a range of plant cell types must be developed for scRNA-seq analysis to become more widespread and accessible to the research community. Recent efforts to apply fixation or to isolate nuclei prior to profiling have shown promise in overcoming these obstacles [91–93].

Analysis of single-cell data is complicated by many factors including the inherent sparsity of the data, amplification biases caused by the minuscule amounts of starting cellular RNA and batch effects that occur during sample processing or library preparation [12,94,95]. While these issues can potentially be addressed using the 1000 or so scRNA-seq analysis tools currently available, it is important to remember that these tools have almost exclusively been developed based on mammalian datasets [96]. There may be as yet unrealised challenges associated with plant single-cell datasets or different analysis modes required, warranting the creation of new tools and databases. Furthermore, transcriptomics studies (bulk and single-cell) are reliant on high-quality genome assemblies and annotations for read mapping, gene-level quantification and downstream analyses [97]. These are currently lacking for the vast majority of plant species, including many medicinal plants, limiting the application of the technologies to more common model species. It is very important that we address this challenge if we are to fully investigate the diversity of plant life and efforts are underway through initiatives such as Genomics for Australian Plants and the Earth Biogenome Project, but a task of such magnitude will take substantial time [98,99].

Metabolomics and proteomics are powerful approaches for understanding the complete biotic state of biological systems, but they are not without challenges [100–102]. Proteins and metabolites cannot be amplified which limits detection sensitivity, a problem that becomes more significant in single-cell analysis [101,103]. As a result, highly efficient extraction techniques are required to isolate metabolites and proteins from single cells [101,102]. Mass spectrometry imaging (MSI) has been useful for spatially resolving cellular metabolomes and proteomes [102,104]. A highlight of this technique is its applicability to whole tissue sections, preserving spatial context and circumventing the need to digest plant cell walls [102]. However, current MSI approaches only cater to a handful of proteins and must be improved to provide a wider coverage of single-cell proteomes [102]. It will also be important to devise novel sample preparation protocols to account for the effect of post-translational modifications (PTMs) which modulate protein function and play crucial roles in a wide range of plant biological processes [105,106]. Single-cell analysis of PTMs remains challenging due to their highly dynamic nature and the considerable amount of starting material required currently for PTM enrichment relative to total protein [102].

The adoption of multi-omics strategies is becoming routine in genomics projects. Earlier studies often analysed ‘omics datasets independently then combined the results to provide better insight into the biological system being studied [107]. However, these processes can be represented more accurately by true integrative analysis of these individual modalities [107–111]. This is challenging at many levels and requires the careful consideration of several factors, such as the computational burden associated with the storage and analysis of various data types and the lack of heterogeneity across ‘omics technologies [107–109]. The increasing interest in single-cell analyses and the tremendous opportunity that single-cell multi-omics represents pose additional analysis challenges such as the preponderance of missing values and noise across modalities [107]. While specialised software and workflows are continuously being developed and refined to address these issues, it is unclear how these will perform with plant single-cell datasets and how they will accommodate the analysis of emerging modalities for plant single-cell research.

## Summary

- Individual cells and tissues are defined by their unique gene regulatory programmes. These drive the characteristic growth, development and physiology of cell types, enabling their particular function within the broader organism.
- Classically most ‘omics approaches in plant biology have focused on bulk tissue samples, achieving organ-level resolution at best. However, recent advances in microfluidic cell handling and spatial analyses now enable transcriptome, proteome and metabolome analysis at a resolution of single to tens of cells.
- Gene regulatory network analyses can be applied to integrate data from these different modalities, enhancing our ability to understand the individual and concerted roles of genes, proteins and metabolites in the biology of cell types.

## Open Access

Open access for this article was enabled by the participation of La Trobe University in an all-inclusive *Read & Publish* agreement with Portland Press and the Biochemical Society under a transformative agreement with CAUL.

## Abbreviations

BIA: benzylisoquinoline alkaloid
CNV: copy number variation
LCM: laser-capture microdissection
MSI: mass spectrometry imaging
PCA: Plant Cell Atlas
PTMs: post-translational modifications
scRNA-seq: single-cell RNA-seq
